# *Saussurea lappa* Clarke-Derived Costunolide Prevents TNF****α****-Induced Breast Cancer Cell Migration and Invasion by Inhibiting NF-**κ**B Activity

**DOI:** 10.1155/2013/936257

**Published:** 2013-08-13

**Authors:** Youn Kyung Choi, Sung-Gook Cho, Sang-Mi Woo, Yee Jin Yun, Jeakyung Jo, Wooyoung Kim, Yong Cheol Shin, Seong-Gyu Ko

**Affiliations:** Laboratory of Clinical Biology and Pharmacogenomics, Center for Clinical Research and Genomics, Department of Preventive Medicine, College of Korean Medicine, Kyung Hee University, 1 Hoegi-dong, Seoul 130-701, Republic of Korea

## Abstract

*Saussurea lappa* Clarke (SLC) has been used as a traditional medicine in Korea, China, and Japan for the treatment of abdominal pain and tenesmus. Costunolide, a sesquiterpene lactone isolated from SLC, has diverse medicinal effects. However, the anticancer effects of costunolide are still unclear in breast cancer. In this study, we demonstrate that costunolide suppresses tumor growth and metastases of MDA-MB-231 highly metastatic human breast cancer cells via inhibiting TNF*α*-induced NF-*κ*B activation. Costunolide inhibited MDA-MB-231 tumor growth and metastases without affecting body weights in the *in vivo* mouse orthotopic tumor growth assays. In addition, costunolide inhibited *in vitro* TNF*α*-induced invasion and migration of MDA-MB-231 cells. Costunolide further suppressed TNF*α*-induced NF-*κ*B signaling activation, resulting in a reduced expression of MMP-9, a well-known NF-*κ*B-dependent gene to mediate breast cancer cell growth and metastases. Therefore, we conclude that SLC and its derivative costunolide suppress breast cancer growth and metastases by inhibiting TNF*α*-induced NF-*κ*B activation, suggesting that costunolide as well as SLC may be promising anticancer drugs, especially for metastatic breast cancer.

## 1. Introduction

Most breast cancer is an epithelial tumor that develops from mammary gland tissue and the inner lining of milk ducts [1]. Metastatic breast cancer is not well cured by surgery, radiotherapy, and chemotherapy [2–4]. Cancer metastasis is the spread of tumor cells from an original site to distant parts of the body. This event consists of multistep processes, which includes tumor cell dissemination, extracellular matrix (ECM) degradation, tumor cell invasion into the ECM, angiogenesis, and secondary metastatic tumor growth [5–7]. Interestingly, primary tumors metastasize to specific organs; for example, aggressive breast cancers selectively metastasize to lung, bone, and brain tissue. This organ tropism seems to be related to different gene expression patterns [8–10].

TNF*α* is frequently detected in many human cancer tissues including breast, ovarian, and renal cancers [11, 12]. In addition, tumor cells producing TNF*α* are correlated with poor prognoses [11]. TNF*α* signaling activation through TNF receptor leads to promoting a recruitment of adaptor proteins and to activating signal cascades including NF-*κ*B pathway [13, 14]. NF-*κ*B regulates diverse physiological and pathological processes including development, metabolism, inflammation, and tissue homeostasis by regulating expression of various genes. In particular, genes regulated by NF-*κ*B play roles such as development, proliferation, survival, and metastasis in cancer [15–17]. NF-*κ*B protein bound to I*κ*B*α* in the cytoplasm is maintained as an inactive state [18]. In response to NF-*κ*B activation signals, IKK*α*/*β* complex is activated, resulting in phosphorylation of I*κ*B*α* on serine residues 32 and 35. Phosphorylated I*κ*B*α* is then ubiquitinated, and polyubiquitinated I*κ*B*α* is degraded through proteasomal pathway. As a result, free NF-*κ*B translocates from the cytoplasm to the nucleus and binds to specific DNA sequences to regulate expression of target genes, which are related to tumor development and metastases [19–21].

The dried root of *Saussurea lappa* Clarke (SLC) has transitionally been used as an ingredient in Korea, China and Japan for the treatment of either abdominal pain or tenesmus. Several earlier studies indicated that the root of SLC has anticancer effect in gastric cancer cells [22, 23]. Costunolide (C_15_H_20_O_2_), a sesquiterpene lactone that is a major component of the root of SLC [24] has been reported to have diverse effects such as anti-inflammatory [25], anti-viral [26], and -fungal [27] effects. Furthermore, costunolide affected anti various cancers including melanoma [28], intestinal [29], leukemia [30], prostate [31], and breast cancers [32]. 

 While anti-cancer effects of either SLC or costunolide have been reported as mentioned befor, antimetastatic effects of either SLC or costunolide on metastatic breast cancer are still poorly understood. In this study, we found that SLC and costunolide inhibit TNF*α*-mediated breast cancer cell migration and invasion by inhibiting NF-*κ*B activation, thereby suggesting the antimetastatic property of costunolide using highly metastatic MDA-MB-231 breast cancer cells. 

## 2. Materials and Methods

### 2.1. Reagents and Cell Lines

Costunolide (molecular weight of 232.32, purity > 99%, see Figure 2(a)) was purchased from Wako (Wako Pure Chemical Industries, Osaka, Japan). RPMI 1640, fetal bovine serum (FBS), antibiotic-antimycotic, and phosphate-buffered Saline (PBS) were purchased from Gibco-BRL (Rockville, MD, USA). EZ-western detection kit was obtained from Daeillab (Daeillab service, Co., Seoul, Korea). TNF*α* was purchased from R&D systems (Minneapolis, MN,USA). 

### 2.2. Preparation of *Saussurea lappa* Clarke (SLC) Extract *Saussurea lappa*

Clarke was purchased from Omniherb (Gyeong Buk, Korea). The 100 g of root of SLC was dipped in 1 L of 80% ethanol and sonicated by using an ultrasonicator (Branson, MO, USA) for 30 min at room temperature. The sonicated extract was filtered through a 0.22 mm filter and concentrated. The ethanol extracts were dried in a 42°C by using a vacuum pump evaporator (Eyela, Tokyo, Japan). The 28.5 g of concentrated extract was dissolved in DMSO to prepare a stock solution of 100 mg/mL. The stock solution was stored at −80°C until use. 

### 2.3. Cell Migration and Invasion

Cell migration was measured by wound healing assays. Cells were seeded in 6-well plates and scratched with a 200 *μ*L pipette tip. 24 hours after treatments with *Saussurea lappa* Clarke and costunolide, migrated cell numbers were counted. For invasion assay, cells were seeded in the upper chambers precoated with Matrigel and treated with SLC and costunolide. Low chambers were filled with 10% FBS or TNF*α*-contained medium, and invasive cells were stained with hematoxylin and eosin to visualize and count. All experiments were performed in triplicate and student's *t*-test was performed to determine statistics. *P* values below 0.05 and 0.001 were considered statistically significant. All data was represented as the mean ± standard deviation. 

### 2.4. Immunofluorescence  Assays

Immunofluorescence assays were used for p-NF*κ*B nuclear translocation in MDA-MB-231 cell. After treatment with SLC and costunolide for 6 hours, cells were fixed with 4% paraformaldehyde for 15 min and then permeabilized with 0.5% Triton X-100 for 10 min. The cells were washed with PBS, blocked with 5% FBS in PBS for 30 min, and then incubated with anti-p-NF-*κ*B antibody overnight at 4°C and with anti-Alexa Fluor-488 secondary antibody (Invitrogen, Eugene, Oregon, USA) for 1 hour. Phalloidin (Sigma) and TO-PRO-3 (Invitrogen) were used to contain F-actin and the nucleus, respectively. Images were obtained with Olympus FV10i Self-Contained Confocal Laser System. The object was 20x, and scale bars on the image indicate 50 *μ*m. 

### 2.5. Luciferase Assays

 Cells were seeded in 24-well plates and NF-*κ*B-luc plasmid (Stratagene, La Jolla, CA, USA) transfected in MDA-MB-231 cells by using Lipofectamine reagent (Invitrogen, Carlsbad, CA, USA). Cells were treated with SLC and costunolide for 6 hours, and then the luciferase assays were done by using dual-luciferase reporter assay (Promega, Madison, WI, USA). All transfections included the RLTK-Luc (kindly provided by Sang Hoon Kim) for transfection efficiency. All experiments were performed in triplicate and student's *t*-test was performed to determine statistics. *P* values below 0.05 and 0.001 were considered statistically significant. All data was represented as the mean ± standard deviation. 

### 2.6. Western Blot

Total protein (30 *μ*g) was separated by SDS-PAGE. After electrophoresis, the proteins were transferred to a nitrocellulose membrane. The membrane was blocked, incubated overnight at 4°C with primary antibodies, washed with PBS-T (PBS with 0.1% Tween-20), and incubated with appropriate HRP-conjugated secondary antibodies at room temperature for 1 hour. Immunoreactive protein was developed using an EZ-western detection kit (Daeillab service, Co., Seoul, Korea). Anti-MMP-9, -p-IKK, -IKK, -p-I*κ*B, -I*κ*B, -p-NF-*κ*B, and -NF-*κ*B were purchased from Cell Signaling (Danvers, MA, USA). Anti-Tubulin was purchased from Sigma (Louis, MO, USA). 

### 2.7. RNA Extraction and RT-PCR

Cellular total RNA was extracted with TRIzol reagent (Invitrogen). The RNA concentration and purity were measured using a spectrophotometer. cDNA was synthesized from total RNA (1 *μ*g) by reverse transcription. The primer sequences and product size were as follows: MMP-9 (262 bp) forward: 5′-CACTGTCCACCCCTCAGAGC-3′, reverse: 5′-GCCACTTGTCGGCGATAAGG-3′, GAPDH (300 bp) forward: 5′-CGTCTTCACCACCATGGAG-A-3′, reverse: 5′-CGGCCATCACGCCACAGTTT-3′. The products were checked by agarose electrophoresis and analyzed using ChemiDoc imaging system (BioRad, Hercules, CA, USA). 

### 2.8. Gelatin Zymography Assay

Conditioned medium was harvested, concentrated, mixed with nonreducing sample buffer, and separated by SDS-PAGE electrophoresis containing 0.1% gelatin. After electrophoresis, the gel was washed with washing buffer (2.5% Triton X-100 in reaction buffer) and then incubated in reaction buffer (50 mM Tris-HCl, 5 mM CaCL_2_, 1 *μ*M ZnCl_2_, and pH 7.4) for 18 h at 37°C. To visualize, the gel was stained with Coomassie brilliant blue R-250 and destained in 50% methanol, 40% distilled water, and 10% acetic acid. 

### 2.9. *In Vivo* Studies

Animal studies were approved by Kyung Hee University Institutional Animal Care and Use Committee (KHU-IACUC). Six-week-old nude (Nu/Nu) mice were purchased from Oriental Science and injected orthotopically into the 4th mammary fat fads with MDA-MB-231 cells (1 × 10^6^ resuspended in a 1 : 1 mixture of PBS and growth factor-reduced matrigel (BD Biosciences, San Jose CA, USA)). A day after tumor cell injection, 20 *μ*M of costunolide was injected into the mammary fat fads three times a week for 30 days. Tumor volumes were measured using calipers and calculated using the following formula: tumor volume (cubic millimeters) = width^2^ × length/2. In addition, body weight was monitored. 

### 2.10. Immunohistochemistry

Tumors were fixed with 4% formaldehyde for further analyses. Tumor tissues were embedded in paraffin, dissected with 5 *μ*m, and deparaffinized in 100% xylene and ethanol series (100%, 95%,and 70%). Heat-induced antigen retrieval was 10 mM sodium citrate buffer for 5 min. Endogenous peroxidase was blocked with peroxidase blocking reagent containing 3.5% hydrogen peroxide. Nonspecific antigen was blocked with serum containing PBS followed by incubation with human ki-67 (5 *μ*g/mL) (Abcam, MA, USA) and MMP-9 antibody (1 : 100) (Cell Signaling, Beverly, MA, USA) overnight at 4°C. It was incubated with biotin-labeled rabbit antibody for 1 hour at room temperature and incubated with ABC and DAB buffer substrate. Sections were visualized with DAB and hematoxylin, mounted, and analyzed using a bright field microscope. The object was 20x, and the scale bars on the image indicate 10 *μ*m. 

### 2.11. Statistics

Data were shown as the means and standard deviations. *P* values less than 0.05 in the two-tailed Student's *t*-test or one-way ANOVA were considered statistically significant.

## 3. Results

### 3.1. *Saussurea lappa* Clarke Suppresses TNF*α*-Induced Breast Cancer Cell Migration and Invasion via an Inhibition of NF-*κ*B Activation

**Figure 1:**
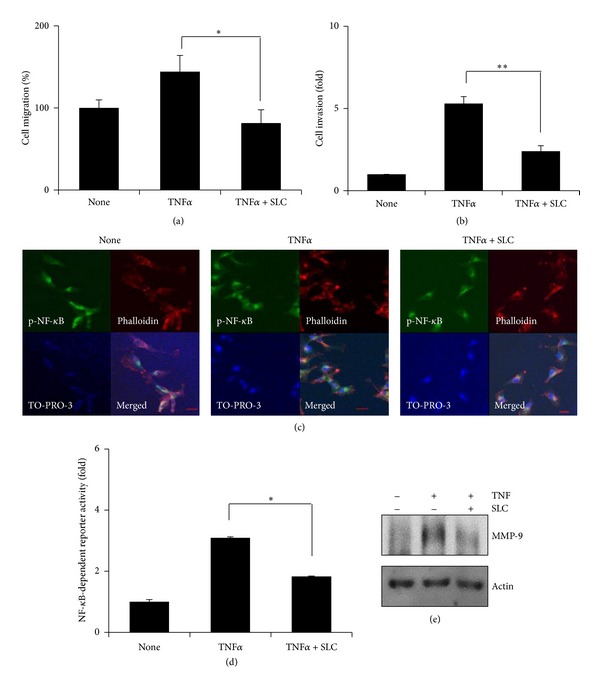
SLC inhibits TNF*α*-induced MDA-MB-231 cell migration and invasion by inhibiting NF-*κ*B activation. (a) Cell migration was measured by wound healing assay. MDA-MB-231 cells were seeded and scratched, pretreated with SLC for 1 hour, and then exposed to TNF*α* for 24 hours. Cell migration was determined by counting cell numbers migrated from the wound healing region. *P < 0.05. (b) MDA-MB-231 cells were seeded on the upper chambers and pretreated with SLC for 1 hour and then exposed to TNF*α* for 24 hours. Invading cells were stained with hematoxylin and eosin, and the cell numbers were measured. **P < 0.001. (c) MDA-MB-231 cells were pretreated with SLC for 1 hour, then exposed to TNF*α* for 6 hours, and stained with p-NF-*κ*B antibody. Phalloidin and TO-PRO-3 were for staining F-actin and the nucleus, respectively. The object was 20x, and scale bars on the image indicate 50 *μ*m. (d) MDA-MB-231 cells were transfected with the NF-*κ*B-dependent luciferase reporter, pretreated with SLC for 1  hour, and then exposed to TNF*α* for 6 hours. Luciferase assays were done by using dual-luciferase reporter assay. All transfections included the RLTK-Luc for transfection efficiency. *P < 0.05. (e) MDA-MB-231 cells were pretreated with SLC for 1 hour and then exposed to TNF*α* for 24 hours. MMP-9 protein was measured by western blotting. Tubulin was used for the loading control.

Because TNF*α* expression is abundant in tumor microenvironment, and its expression is correlated with poor prognoses [14, 15], we investigate effects of *Saussurea lappa* Clarke (SLC) on highly metastatic MDA-MB-231 cells. In normal culture condition, SLC treatment (50 *μ*g/mL) inhibited MDA-MB-231 cell migration (data not shown). Next, TNF*α* increased the MDA-MB-231 cells migration compared to nontreated cells, and 50 *μ*g/mL of SLC suppressed TNF*α*-induced MDA-MB-231 cells migration by approximately 63% (Figure 1(a)). In addition, 50 *μ*g/mL of SLC significantly inhibited TNF*α*-induced cell invasion (Figure 1(b)). Next, to clarify the mechanism of SLC to inhibit cell migration and invasion, we performed the immunofluorescence assays to examine NF-*κ*B pathway. As shown in Figure 1(c), TNF*α* induced nuclear translocation of phosphorylated NF-*κ*B, which was blocked by SLC. In the luciferase assay, SLC inhibited TNF*α*-induced NF-*κ*B-dependent transcriptional activity (Figure 1(d)). Accordingly, 50 *μ*g/mL of SLC suppressed TNF*α*-induced mRNA and protein expression of MMP-9 that is well known as NF-*κ*B-dependent gene (Figure 1(e)). Thus, our data indicate that SLC inhibits TNF*α*-induced highly metastatic MDA-MB-231 human breast cancer cell migration, invasion, and NF-*κ*B activation. 

### 3.2. *Saussurea lappa* Clarke-Derived Costunolide Suppresses Cell Migration and Invasion in Breast Cancer Cells

**Figure 2:**
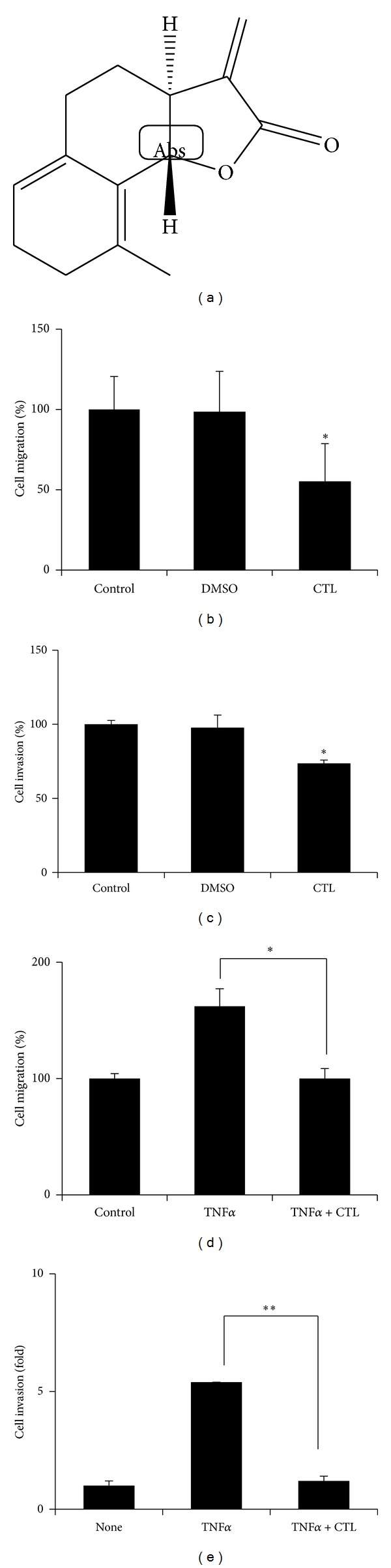
Costunolide inhibits TNF*α*-induced MDA-MB-231 cell migration and invasion. (a) Structure of costunolide. (b) MDA-MB-231 cells were seeded and scratched. Treatment with costunolide for 24 hours and counted. *P < 0.05. (c) MDA-MB-231 cells were seeded on the upper chambers and treated with costunolide in 0% serum. Low chamber filed with 10% serum. *P < 0.05. (d) Pretreated with costunolide for 1 hour and then exposed to TNF*α* for 24 hours. Cell migration was determined by counting numbers of cells migrated from the wound healing region. *P < 0.05. (e) MDA-MB-231 cells were seeded on the upper chambers and treated with costunolide. Low chamber filed with TNF*α*. *P < 0.001.

Since Costunolide (C_15_H_20_O_2_) is a major component of SLC [22], we examined whether SLC-derived costunolide inhibits metastatic properties of breast cancer cells. Costunolide (20 *μ*M) blocked cells migration in normal serum conditions by approximately 45% (Figure 2(a)). Next, to examine costunolide effect on cells invasion, MDA-MB-231 cells were seeded in the upper chambers precoated with matrigel and treated with costunolide in 1% serum contained media, and the low chambers were filled with 10% serum contained media. As shown in Figure 2(b), a treatment of breast cancer cells with 20 *μ*M of costunolide for 24 hours reduced cells invasion. 

Next we performed experiments to determine whether costunolide inhibits TNF*α*-induced cells migration and invasion. 20 *μ*M of costunolide suppressed TNF*α*-induced MDA-MB-231 cell migration by approximately 62% (Figure 2(c)). In addition, whereas TNF*α* increased an invasiveness of MDA-MD-231 cells by approximately five folds, costunolide significantly inhibited TNF*α*-induced cell invasion by approximately five folds (Figure 2(d)). 

### 3.3. *Costunolide Inhibits *NF-*κ*B Pathway in Breast Cancer Cells

**Figure 3:**
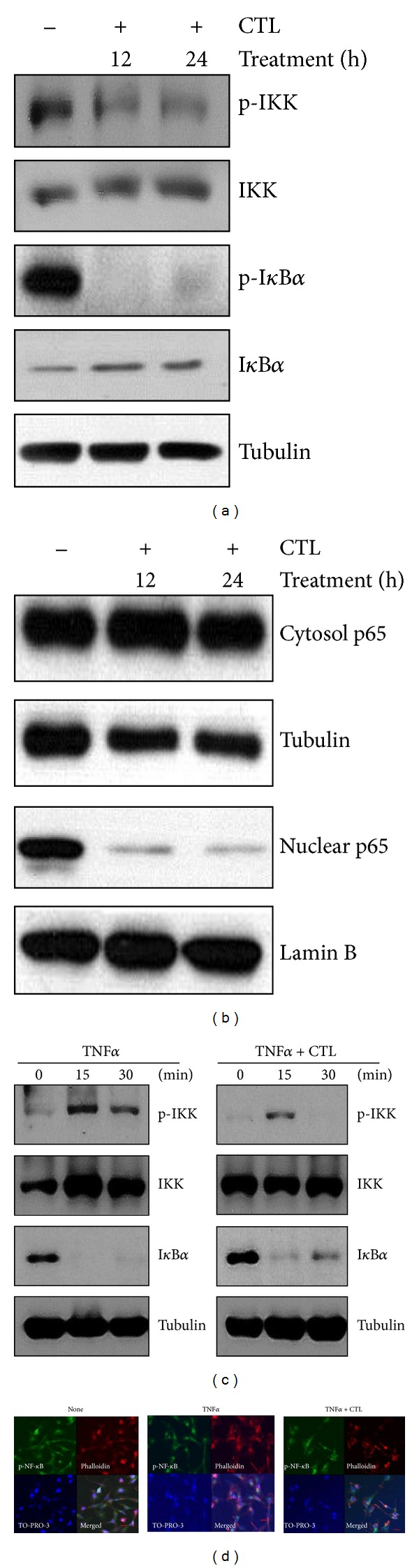
Costunolide inhibits TNF*α*-induced NF-*κ*B pathway in MDA-MB-231 cells. (a) MDA-MB-231 cell were treated with costunolide for indicated time periods. Whole lysates were analyzed by western blotting with anti-pIKK, -IKK, -pI*κ*B*α*, -I*κ*B*α*, and Tubulin. (b) Cells were fractionated into cytoplasmic and nuclear compartment and western blotting for NF-*κ*B. Tubulin and LaminB were used as loading control. (c) MDA-MB-231 cells were treated with TNF*α* and cotreated with TNF*α* and costunolide for 15 to 30 min. Whole lysates were analyzed by western blotting with anti-pIKK, -IKK, -I*κ*B*α*, and Tubulin. (d) MDA-MB-231 cells were pretreated with costunolide for 1 hour, then exposed to TNF*α* for 6 hours, and stained with p-NF-*Κ*b antibody. Phalloidin and TO-PRO-3 were for staining with F-actin and nucleus, respectively. The object was 20x, and the scale bars on the image indicate 50 *μ*m.

Next we examined costunolide effect on NF-*κ*B signaling pathway in MDA-MB-231 cells. As shown in Figure 3(a), costunolide inhibited phosphorylation of IKK and I*κ*B*α*, resulting in blocking I*κ*B*α* degradation in a time-dependent manner. Accordingly, a treatment of the MDA-MB-231 cells with costunolide inhibited the nuclear translocation of p65 NF-*κ*B subunit (Figure 3(b)). 

Next, in order to examine whether costunolide suppresses TNF*α*-induced NF-*κ*B pathway, we stimulated cells with TNF*α* for 15 to 30 min in the presence or absence of costunolide. As shown in Figure 3(c), TNF*α*-induced IKK phosphorylation was prolonged until 30 min, which was blocked by costunolide. Furthermore, while TNF*α* induced I*κ*B degradation, costunolide slowly recovered I*κ*B*α* expression at 15 min. 

To confirm costunolide suppression of NF-*κ*B nuclear translocation, we performed immunofluorescence assay using the anti-pNF-*κ*B antibody. As shown in Figure 3(d), NF-*κ*B was observed in the cytosol of the cells treated with costunolide. Thus, our data indicate that SLC-derived costunolide inhibits NF-*κ*B pathway. 

### 3.4. *Costunolide Inhibits *NF-*κ*B Transcriptional Activity and MMP-9

**Figure 4:**
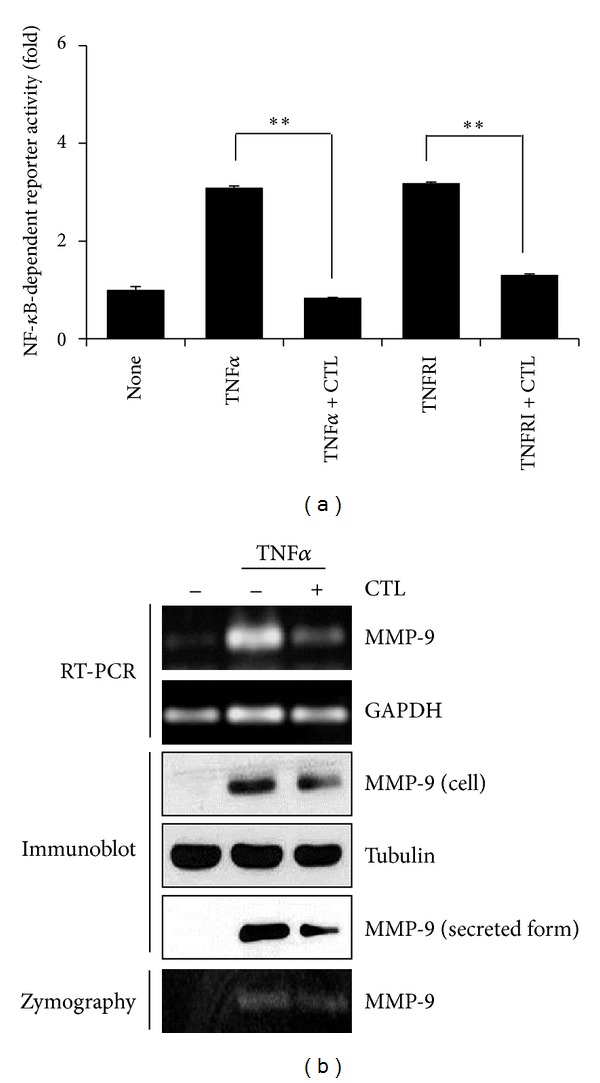
Costunolide inhibits TNF*α*-induced NF-*κ*B activity and MMP-9 expression. (a) MDA-MB-231 cells were transfected with the NF-*κ*B-dependent luciferase reporter, pretreated with costunolide for 1 hour, and then exposed to TNF*α* for 6 hours. In addition, after cotransfected with TNFRI and NF-*κ*B-dependent luciferase reporter, treated with costunolide for 6 hours. Luciferase assay were done by using dual-luciferase reporter assays. All transfections included the RLTK-Luc for transfection efficiency. **P < 0.001. (b) MDA-MB-231 cells were pretreated with costunolide for 1 hour and then exposed to TNF*α* for 6 hours. MMP-9 expression was analyzed by RT-PCR, western blotting, and zymography.

To confirm the inhibition of NF-*κ*B pathway by costunolide, we performed the transcriptional activation of NF-*κ*B by using the luciferase assay. As shown in Figure 4(a), costunolide reduced TNF*α*-induced NF-*κ*B transcriptional activation by 5-fold in MDA-MB-231 cells. We next examined whether costunolide affects upstream of IKK in TNF*α*-induced NF-*κ*B pathway, MDA-MB-231 cells were cotransfected with NF-*κ*B reporter gene and TNFRI and then cultured in the presence or absence of costunolide. Costunolide reduced TNFRI-induced NF-*κ*B transcriptional activity by approximately 2.5-fold in MDA-MB-231 cells (Figure 4(a)). 

It is known that MMP-9 is regulated by NF-*κ*B, and the promoter region of MMP-9 gene contains binding sites for NF-*κ*B. Thus, we examined whether costunolide inhibits MMP-9; we checked MMP-9 by using RT-RCR, western blot, and zymography. As shown in Figure 4(b), Costunolide inhibited TNF*α*-induced MMP-9 mRNA, protein, and enzyme activity, when cells were treated with costunolide for 24 hours. 

### 3.5. Costunolide Inhibits Tumor Growth and Metastasis

**Figure 5:**
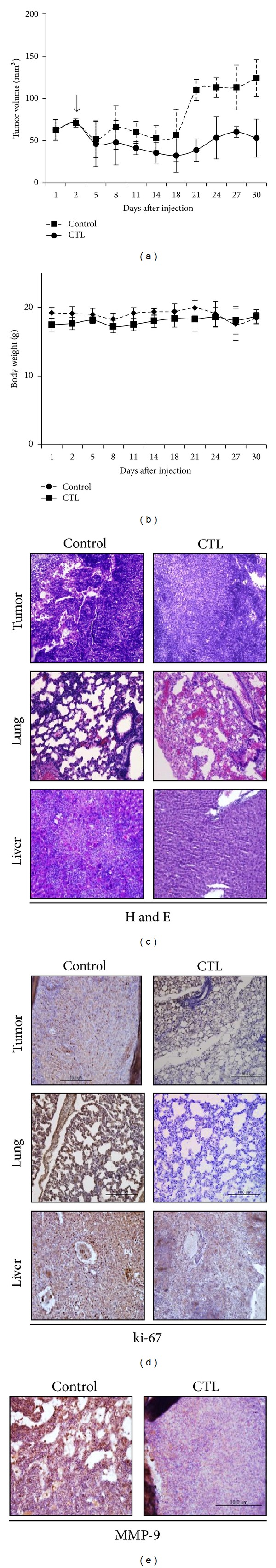
Costunolide inhibits orthotopically tumor growth and metastasis. (a) 1 × 10^6^ MDA-MB-231 cells were orthotopically injected in nude mice (*n* = 5/group). Costunolide was injected into the mammary fat fad and repeated every three days for 30 days. Tumor volumes were measured using calipers. Tumor volume (cubic millimeters) = width^2^ × length/2. (b) Body weight measured three times a week. (c) Tumor tissues were stained with hematoxylin and eosin. Photo images were taken at 20x magnification. (d) Tumor tissues were stained with anti-ki-67 antibody. (e) Tumor tissues were stained with anti-MMP-9 antibody. The object was 20x, and, scale bars on the image indicate 10 *μ*m.

To examine costunolide effect on breast cancer growth and metastases *in vivo*, MDA-MB-231 cells were orthotopically injected into the 4th mammary fat fads. A day after tumor cell injection, costunolide at 20 *μ*M was injected into the mammary fat fad three times a week for 30 days. In addition, tumor volume and body weight of mice were also measured three times a week. As shown in Figure 5(a), costunolide reduced tumor volume (*P* = 0.007628), and no significant weight loss in mice treated with either costunolide or vehicle was observed (Figure 5(b)). When tumor tissues were stained with hematoxylin and eosin, we found that tumor cohort treated with costunolide compared to that with control was well differentiated (Figure 1(c)). In addition, tumor and organ (lung and liver) tissues were stained with anti-ki-67. Costunolide, compared to control reduced ki-67 positive cell in tumor, lung, and liver (Figure 1(d)). When tumor tissues were stained with MMP-9 antibody, costunolide inhibited a number of MMP-9 positive cells (Figure 1(e)). Thus, our data indicate that costunolide inhibits tumor growth and metastasis. 

## 4. Discussion

TNF*α*-induced NF-*κ*B pathway is a well-known molecular target for cancer therapy. Tumor cells released NF-*κ*B-dependent MMPs by NF-*κ*B-mediated TNF*α* production of immune cells in tumor microenvironment [33]. In this study, we found that *Saussurea lappa* Clarke-derived costunolide suppressed TNF*α*-induced MDA-MB-231 breast cancer cell migration and invasion by inhibiting NF-*κ*B activity (Figure 5(c)). Thus, SLC as well as costunolide appears to be useful for treating highly metastatic breast cancer. 

 Matrix metalloproteinases (MMPs), a family of zinc-dependent endoproteinase is necessary for extracellular matrix (ECM) degradation among metastasis process. MMPs also affect many biological processes such as normal tissue remodeling, wound healing, angiogenesis, embryogenesis, and many diseases including cancer, atheroma, and arthritis [34]. MMP-9 is frequently overexpressed in many cancers and correlates with poor prognosis and survival in cancer patients [35–37]. In addition, MMP-9 is important for tumor metastasis by cleaving basement membranes, which allows migratory phenotype cells to be more invasive and motile [38–40]. MMP-9 is regulated by stimulators (phorbol 12-myristate 13-acetate, PMA; transforming necrosis factor alpha, TNF*α*; growth factor, UV; and stress) and transcription factors (nuclear factor kappaB; NF-*κ*B and activator protein-1; AP-1) [39, 40]. In addition, MMP-9 is important for tumor metastasis by cleaving basement membranes, which allows migratory phenotype cells to be more invasive and motile. Thus, TNF*α*-induced MMP-9 expression via NF-*κ*B is important for cancer growth and metastasis. In our study, TNF*α*-induced cell migration and invasion were inhibited by either SLC or costunolide. SLC and costunolide suppressed TNF*α*-induced NF-*κ*B translocation to nucleus and transcriptional activity. In addition, costunolide specifically inhibited IKK phosphorylation and I*κ*B*α* degradation. Those inhibitions further reduced NF-*κ*B-dependent MMP-9 expression. As a result, costunolide suppressed *in vivo* tumor growth and metastasis. 

**Figure 6:**
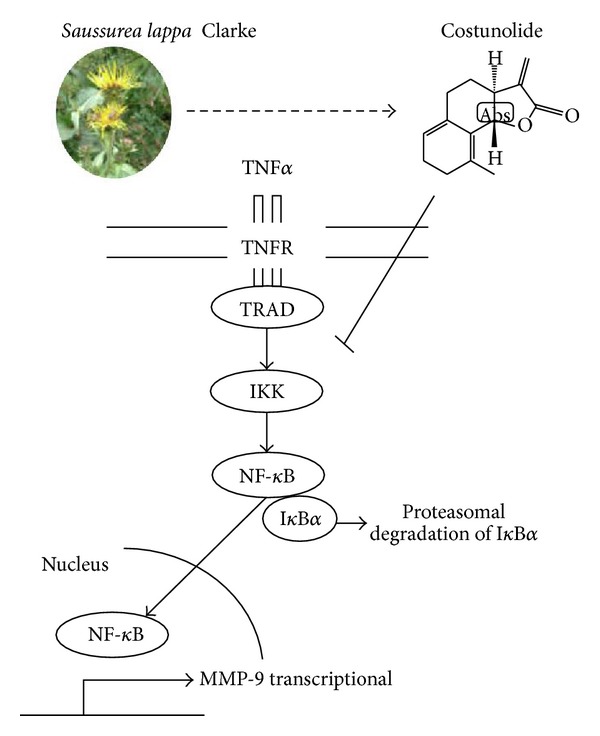
Schematic representation of the mechanism where costunolide inhibits TNF*α*-induced breast cancer cell migration and invasion by inhibiting NF-*κ*B activity.

 This study concludes that (a) SLC suppresses TNF*α*-induced MDA-MB-231 cell migration and invasion by inhibiting NF-*κ*B-dependent MMP-9 expression, (b) SLC-derived costunolide inhibited serum or TNF*α*-induced MDA-MB-231 cell migration and invasion, (c) costunolide inhibited TNF*α*-induced NF-*κ*B translocation resulting from the suppression phosphorylation and I*κ*B*α* degradation (d) costunolide blocked TNF*α*-induced NF-*κ*B transcription activity and TNF*α*-induced MMP-9 expression, and (e) costunolide decreased *in vivo *tumor growth and metastasis without weight loss (Figure 6). In sum, we provide evidence that the anti-cancer effect of both SLC and its component costunolide on MDA-MB-231 result from the inhibition of TNF*α*-induced NF-*κ*B activation. Therefore, SLC-derived costunolide could be useful for treating highly metastatic breast cancer growth and metastases.
